# Space use and habitat selection of an invasive mesopredator and sympatric, native apex predator

**DOI:** 10.1186/s40462-020-00203-z

**Published:** 2020-05-04

**Authors:** Michael L. Wysong, Bronwyn A. Hradsky, Gwenllian D. Iacona, Leonie E. Valentine, Keith Morris, Euan G. Ritchie

**Affiliations:** 1 School of Biological Sciences, University of Western Australia, Crawley, Perth, WA 6009 Australia; 2 Present Address: Nyamba Buru Yawuru, 55 Reid road, Cable Beach, WA 6726 Australia; 3 School of Biosciences, The University of Melbourne, Parkville, VIC 3010 Australia; 4 Australian Research Council Centre of Excellence for Environmental Decisions, School of Biological Sciences, The University of Queensland, St. Lucia, QLD 4072 Australia; 5 Biodiversity and Conservation Science, Department of Biodiversity, Conservation and Attractions, Woodvale, WA 6946 Australia; 6 Centre for Integrative Ecology, School of Life and Environmental Sciences, Deakin University, Burwood, VIC 3125 Australia

**Keywords:** Dingo (*Canis dingo*), Feral cat (*Felis catus*), GPS tracking, Habitat selection, Home range, Kernel density estimation, Movement ecology, Predator interaction, Step selection function

## Abstract

**Background:**

Where mesopredators co-exist with dominant apex predators, an understanding of the factors that influence their habitat and space use can provide insights that help guide wildlife conservation and pest management actions. A predator’s habitat use is defined by its home range, which is influenced by its selection or avoidance of habitat features and intra- and inter-specific interactions within the landscape. These are driven by both innate and learned behaviour, operating at different spatial scales. We examined the seasonal home ranges and habitat selection of actively-managed populations of a native apex predator (dingo *Canis dingo*) and invasive mesopredator (feral cat *Felis catus*) in semi-arid Western Australia to better understanding their sympatric landscape use, potential interactions, and to help guide their management.

**Methods:**

We used kernel density estimates to characterise the seasonal space use of dingoes and feral cats, investigate inter- and intra-species variation in their home range extent and composition, and examine second-order habitat selection for each predator. Further, we used discrete choice modelling and step selection functions to examine the difference in third-order habitat selection across several habitat features.

**Results:**

The seasonal home ranges of dingoes were on average 19.5 times larger than feral cats. Feral cat seasonal home ranges typically included a larger proportion of grasslands than expected relative to availability in the study site, indicating second-order habitat selection for grasslands. In their fine-scale movements (third-order habitat selection), both predators selected for roads, hydrological features (seasonal intermittent streams, seasonal lakes and wetlands), and high vegetation cover. Dingoes also selected strongly for open woodlands, whereas feral cats used open woodlands and grasslands in proportion to availability.

**Management recommendations:**

Based on these results, and in order to avoid unintended negative ecological consequences (e.g. mesopredator release) that may stem from non-selective predator management, we recommend that feral cat control focuses on techniques such as trapping and shooting that are specific to feral cats in areas where they overlap with apex predators (dingoes), and more general techniques such as poison baiting where they are segregated.

## Background

Apex predators can shape the composition and function of ecosystems through their effects on sympatric competitors and prey [1]. By suppressing herbivore populations [2], or affecting the distribution and space use of subordinate mesopredators [3–5], apex predators may initiate trophic cascades, with substantial impacts on prey assemblages, ecosystem structure and hence biodiversity [1, 2, 6]. Where mesopredators co-occur with apex predators, they may modify their behaviour to minimise interactions with larger predators [7].

Invasive mesopredators pose a major threat to biodiversity worldwide [8, 9]. In ecosystems where native apex predators occur in sympatry with invasive mesopredators, maintaining the ecological function of native apex predators while mitigating the impacts of invasive mesopredators presents a key challenge for managers. A better understanding of the factors that influence the space use of sympatric predators can therefore help inform more targeted and effective conservation, and pest and wildlife management [10–12].

The space required by an individual predator to survive and reproduce is affected by its interactions with sympatric species and local resource availability [13, 14]. The resulting area, commonly known as the home range, is a fundamental metric in animal ecology [15]. Among predator species, home range area typically scales positively with body size as a result of increasing energetic requirements and specialization on larger prey [16]. However, within a predator population, there is often considerable variation in home range size due to factors including body size, sex, age, reproductive status and season [17]. Therefore, a comparison of space use among sympatric predator species is important for understanding community-level effects, while an examination of space use within a species’ population can provide insights into the species’ ecology, demography and population dynamics.

An individual’s home range is influenced by its selection or avoidance of habitat features within the landscape. This is a hierarchical, multi-scale process which involves a series of innate and learned behavioural decisions that can affect fitness [18]. Broad-scale (second-order) habitat selection describes the selection of a home range from the broader geographic range of a species, and is largely driven by the availability and distribution of resources, climate and landscape features [19]. Finer-scale (third-order) habitat selection describes the use of resources within an individual’s home range, and may be driven by both biotic and abiotic factors [19, 20].

The biological and physical attributes of an environment can influence how predators select habitats and interact with sympatric species. For example, apex predators may preferentially select certain vegetation types according to the likelihood of encountering prey [21–23]. Mesopredators may select vegetation types based on prey availability, but may also alter their movement and activity patterns within landscapes according to the occurrence of larger predators [24, 25]. Linear features such as roads, railway lines and seismic exploration lines can facilitate predator access to prey [26] or influence predator behaviour and patterns of movement [27–29]. Apex predators [30, 31] and mesopredators [32, 33] have been well-documented using roads independently, but there is little understanding of how they use roads when in sympatry.

Topography and vegetation cover can also mediate predator movement and interactions, as predators may select for areas that don’t impede movement and conserve energy, such as gentle contours and more open habitats [34, 35]. Areas of gentle topography are often associated with hydrological features, so movement through these areas may also relate to the presence of water, dense vegetation cover, and/or higher prey availability. Depending on the predator’s hunting mode (e.g. ambush vs. pursuit), physiological constraints and body-size, a predator may select dense vegetation cover to improve hunting success [36], find shade to assist thermoregulation [37], or provide concealment from other predators or humans [38].

Australia provides a highly tractable system in which to study space use and habitat selection of sympatric terrestrial apex- and meso-predators, because of the continent’s relatively simple mammalian predator guild. There is one large (> 10 kg), terrestrial, native apex predator, the dingo (*Canis dingo*) [39], and two recently introduced mesopredators: the feral cat (*Felis catus*) and the European red fox (*Vulpes vulpes*) (L.), with considerable, though not complete, overlap in their mainland distributions. In some regions, native quolls, varanids and large snake species also constitute important terrestrial mesopredators [40]. Introduced predators, particularly feral cats, are responsible for considerable biodiversity loss worldwide [8, 41] and reducing their impacts is a primary goal for the conservation of Australian fauna [9, 42]. As the apex predator, dingoes are predicted to suppress the activity or abundance of feral cats and there is some empirical evidence to support this [43–46], but other evidence is equivocal [47], and the overall extent of top-down suppressive control remains contentious [48, 49].

We examined the seasonal home ranges and habitat selection of a sympatric native apex predator (dingo) and an invasive mesopredator (feral cat) in semi-arid Australia. Most dingo populations in Australia are disrupted, to varying degrees, via lethal control [50]. Poison baiting may be targeted specifically at dingoes or affect dingoes as non-targets. In our landscape, poison baiting for feral cats occurs at the study site and dingoes are controlled on surrounding pastoral properties; these programs are likely to affect the demography and space use of both species. Our objective was to understand the landscape use of dingoes and feral cats and their potential interactions under these conditions to help improve feral cat management while minimising impacts to dingoes.

We characterized home range extent and composition of the two predators to quantify inter- and intra-species variation and examine interspecific differences in broad-scale habitat selection. We then used discrete choice modelling to examine differences in fine-scale habitat selection within their home ranges across several habitat features. We predicted that:

1. Dingoes, as the larger predator, would have larger home ranges than feral cats [51].
2. Like many mammalian predators [52], males of both species would have larger home ranges than females.
3. At a broad scale (second-order habitat selection), dingoes would selectively locate their home ranges in areas with more open woodland, while feral cats would select for grassland based on the availability of preferred prey types in these two habitats: large macropods in woodlands [53] and abundance of small vertebrates in grasslands [54].
4. Within their home ranges (third-order habitat selection), dingoes would select for open woodlands [53], roads [50, 55] and hydrological features [56, 57], consistent with previous research. In contrast, feral cats would avoid these features within their home ranges to minimise interaction with dingoes [44] and because their physiology means they do not require water for drinking [58].
5. Within their home ranges (third-order habitat selection), dingoes would also select for low vegetation cover and feral cats would select for high vegetation cover based on their typical prey and open versus ambush hunting modes [40].

We use the results from our study to make management recommendations for more strategic and effective control of feral cats in the presence of dingoes, where it may be desirable to preserve the ecological function of the larger predator [46] — a challenge common to many ecosystems [12]. By identifying where invasive mesopredators are likely to either overlap or segregate spatially with apex predators, control techniques can be adapted to specifically target invasive mesopredators in areas where they overlap with apex predators and more general techniques applied where they are spatially segregated.

## Methods

### Study area

**Fig. 1:**
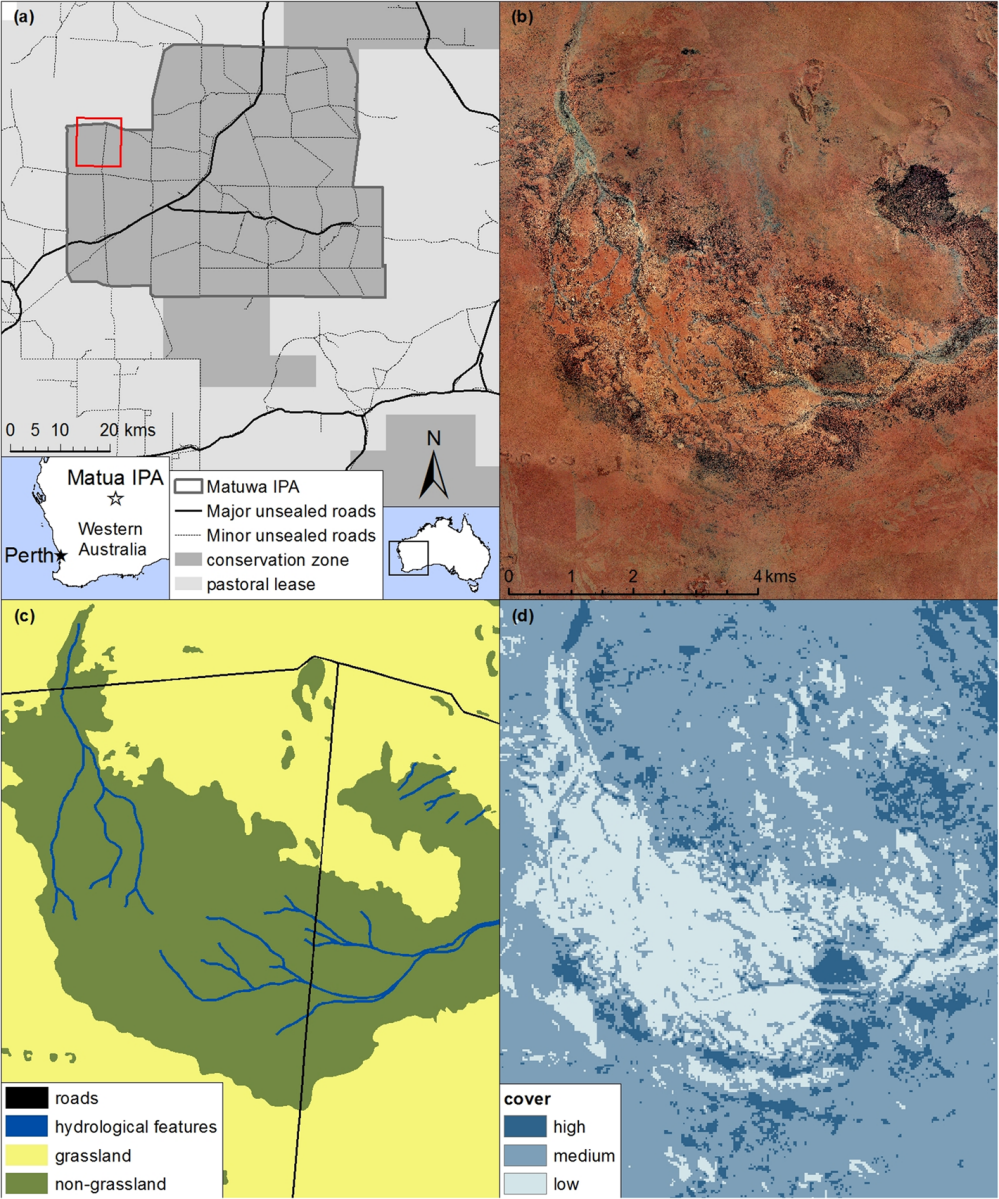
**a** Location of Matuwa Indigenous Protected Area (IPA) and surrounding properties in semi-arid Western Australia. Fine-scale habitat maps for this study were created from high-resolution aerial imagery **b** to delineate features predicted to influence dingo and feral cat habitat selection **c**. Vegetation cover **d**, also a predictor of habitat selection, was mapped at 3 broad classes using a cover index derived from Landsat data recorded during the period of study. (The red box in **a** shows the extent in maps **b-d**)

The study was conducted on the Matuwa Indigenous Protected Area (IPA) and surrounding properties in central Western Australia, 180 km east-northeast of Wiluna (26.23°S, 121.56°E; Fig. 1a). Matuwa IPA is a 2410 km^2^ former pastoral lease that has been managed jointly as a conservation reserve by the Wiluna Aboriginal community and the Western Australia Department of Biodiversity Conservation and Attractions (formerly Parks and Wildlife) since 2000. All cattle (and a few remaining sheep) were removed from the property by 2003 and recursions are largely prevented by an electric cattle fence along the boundary. This fence presents no barrier to the movement of other medium- to large-sized mammal species in the area including dingoes, feral cats and kangaroos. Camels, donkeys and horses occasionally penetrate the fence and are controlled opportunistically. Surrounding land uses include active pastoral leases as well as unallocated crown land which receives little to no active management. Dingoes and feral cats are common throughout the study area, as are other native predators including large varanids and snakes. Non-native European red foxes are uncommon.

The climate of the region is classified as a hot, semi-arid desert with highly erratic and unreliable rainfall. The landscape is dominated by gently undulating sand and rocky plains with stony plateau uplands and breakaways, as well as occasional salt lakes with fringing saline alluvial plains [59]. Vegetation communities can be broadly classified as either open woodlands or open hummock grasslands with occasional samphire shrublands. Open woodlands are frequently dominated by *Acacia aneura* and *Eucalyptus kingsmillii* and occur on rocky plains, stony uplands, and breakaways. Hummock grasslands are dominated by spinifex grasses (*Triodia basedoweii* and *T. melvillei*) with occasional trees and are restricted primarily to the open sand plains. Samphire shrublands typically surround salt lakes and are dominated by halophytic forbs and shrubs.

Feral cat control occurs regularly at Matuwa IPA for the protection of threatened fauna while dingo control occurs intermittently on some of the surrounding pastoral leases for livestock protection. The intensity and regularity of dingo control varies from year to year and between properties. This type of uncoordinated and varying control effort may provide short-term relief from stock depredation and disrupt dingo social stability [60], but is unlikely to have a significant impact on dingo abundance over the long term [61]. A sustained feral cat control program on Matuwa IPA has been in place since 2003 consisting of an annual aerial deployment of toxic sodium monofluroacetate (1080) *Eradicat*® baits in mid-July (winter) at a density of 50 baits km^− 2^. In semi-arid regions of Australia, feral cat baiting is most effective during winter when the availability of prey, such as rabbits and reptiles, is typically low and hence bait uptake is generally greater [9].

The annual baiting regime at Matuwa IPA has reduced feral cat activity – as measured by track count indices – to approximately half that of pre-baiting levels; however the impact on feral cat abundance remains unclear [62]. Generally, feral cat activity drops substantially in the first months following baiting and is highest immediately prior to baiting [62]. Dingoes are also susceptible to *Eradicat*® baits [63], but track indices of dingo activity generally returns to near pre-baiting levels within several months of baiting. This suggests that the scale and frequency of baiting is not enough to outweigh re-invasion by dingoes [64]. Our study was focused on the periods of up to 70 days immediately prior to annual *Eradicat* bait application to take advantage of the period when cat activity was likely to be the highest, and dingoes would be least affected by baiting [65]. Baits were deployed over a four-day period beginning on July 6 in 2013 and July 10 in 2014; any data collected after the start of these baiting periods were excluded from the analysis.

### Animal capture and data collection

Dingoes and feral cats were captured between June 2013 and June 2014 using soft-jaw leg-hold traps (Oneida Victor® Soft Catch® traps, size 1.5 for feral cats, and size 3 for dingoes, Oneida Victor Inc. Ltd., Euclid, USA). Traps were set along unsealed roads at Matuwa IPA and baited with *pongo* [66] (a blended mixture of cat faeces and urine) for feral cats, and with domestic dog faeces and urine for dingoes. Cats were sedated prior to processing. For cats captured in 2013, sedation was achieved via an intramuscular injection of 4 mg/kg Zoletil 100 (100 mg/mL; teletamine-zolazepam). However, a drawback of this procedure was the relative long time – 30 to 60 min – needed for recovery; therefore, subsequent captures used intramuscular injections of 0.05 mg/kg Domitor (1 mg/mL medetomidine) with the reversal agent Antisedan (5 mg/mL atipamazole) administered intramuscularly at half dose upon completion of processing. Dingoes were not sedated but instead were processed following guidelines set by the Department of Agriculture and Food of Western Australia [67]. This procedure employs a catchpole and restraining board to restrain animals prior to processing.

Captured animals that were large enough, based on a maximum 5% collar to body weight ratio (> 1.9 kg cats, > 9.5 kg dingoes), were fitted with Global Positioning System (GPS) collars. Cat GPS collars (Sirtrack Minitrack with VHF download and internal timed release, Havelock North, New Zealand) weighed 120 g and were programmed to drop off after 3 months. Dingo GPS collars (Sirtrack Pinnacle Lite with VHF transmitter, remote download, and external timed release) weighed 475 g and were programmed to drop off after 12 months. Cat collars recorded the location at a minimum of 4-h intervals starting immediately upon release. Dingo collars recorded locations at 2-h intervals starting on the first full hour following release. Locations collected within the first 24 h of release were discarded from analysis.

### Map of landscape features

We developed a fine-scale habitat map of the study area based on four landscape features that we considered likely to influence dingo and feral cat habitat selection or movement: roads, hydrological features, vegetation type and vegetation cover. We digitized the first two features from high-resolution, 80-cm pixel aerial imagery of the study area. All roads in the study site are unsealed and are 5–10 m wide. Hydrological features included seasonal intermittent streams, seasonal lakes and wetlands. Using ArcGIS (v.10.2 ESRI, Redlands, California), we created buffers of 10 m around roads and 20 m around hydrological features in order to encompass errors in the precision of mapping these features; a larger buffer is needed around streams because their mapping is inherently less precise. We mapped two broad classes of vegetation type – grassland and open woodland – by digitizing aerial imagery (Fig. 1b-c). Samphire shrublands represent a relatively low proportion of the vegetation and were not always easy to discern from open woodlands in the imagery, and so we included them within the open woodland classification.

We classified vegetation cover as low, moderate, or high using a Landsat-based vegetation cover index developed by the Land Monitor Project of Western Australia [68]. The index uses spectral bands 3 and 5 to assign a cover value at the 30 m^2^ pixel scale. We applied a Jenks optimization [69] to find the natural breaks in the distribution of these pixel values across the study area to produce three cover classes (Fig. 1d).

In the final habitat map that we constructed of the study landscape, roads represented less than 0.5% of the total area, while hydrological features represented 3.8%, grasslands 19.0% and open woodlands 81.0%. Low cover represented 15.2%, moderate cover 46.4% and high cover 38.4% of the total area. For the purposes of the broad-scale habitat selection analysis (Prediction 3), habitat classifications were simplified as grassland and woodland (all other habitats).

### Data screening and organization

As described above, data analysis was restricted to the periods up to 70 days immediately prior to the annual *Eradicat®* bait application in early July 2013 and 2014. This period also avoided dingo whelping which typically occurs in mid-July [57]. GPS data were screened for positional errors and unrealistic fixes removed based on the species’ known movement speeds [70]. We excluded fixes exceeding a 16 kmh^− 1^ gallop speed and 8.75 kmh^− 1^ trotting speed for dingoes [71] and a 3.2 kmh^− 1^ gallop and 2.0 kmh^− 1^ trotting speed for cats [72]. We also removed fixes with elevation values that were ± 100 m from site reference elevation. Based on field-tested errors, we classified dingo fixes that were > 20 m from the preceding fix, and cat fixes that were > 35 m from the preceding fix, as ‘moving’, as fewer than 5% of the fixes from stationary collar tests exhibited distances larger than these cut off values, respectively. Fixes that did not meet the criterion of ‘moving’ were removed from the analysis as we were only interested in habitat selection when individuals were active.

### Identification of seasonal home ranges

We used fixed kernel density estimation (KDE) to generate seasonal home ranges for each collared animal by producing a probabilistic model of space use defined by a utilisation distribution (UD). We calculated the UD for each individual at the 95 and 50% isopleths to define seasonal home range and seasonal core home range respectively [73] and use the term ‘seasonal’ to reflect that these home range estimations are restricted to the Martu season of *wantajarra* (late autumn – winter) [74]. We chose the reference bandwidth to determine the level of smoothing for KDE calculations because this non-parametric method generally results in a smoother contour with less variability and so presents a more generalized snapshot of space use [75]. Kernel estimates were calculated in R in the package rhr [76]. To determine whether seasonal home ranges were sufficiently described we used asymptote analysis with data added randomly at 15-fix intervals for cats and 25-fix intervals for dingoes. The smaller interval for cats was used because the total number of fixes for feral cats was generally lower than for dingoes since collars on cats were programmed to take half as many fixes as dingoes in a 24-h period. We considered home ranges sufficiently described when 75–100% of the fixes were within 95% of the total KDE area using all the fixes.

### Home range size

We used linear models to test the effects of species on the area of seasonal home range and seasonal core home range (Prediction 1). Data were log_10_-transformed to improve normality and the homoscedasticity of residuals. We also used linear models to test for the effects of sex on seasonal home range area and seasonal core home range area in dingoes (Prediction 2); we were unable to test this prediction for feral cats due to the small number of female cats (*n* = 3). In the latter models, seasonal home range areas were log_10_ transformed for the same reasons as above, but seasonal core home range areas did not require transformation.

### Broad-scale habitat selection

To assess whether dingoes and feral cats selectively located their home ranges in relation to broad habitat types (Prediction 3), we compared the proportion of vegetation type (woodland for dingoes and grassland for feral cats) within observed seasonal home ranges (“used”) to the proportion of that vegetation type within randomly-placed circular home ranges (“available”, *n* = 1000) equal in area to the median seasonal home range (48,325 ha for dingoes, 2482 ha for feral cats) [77, 78]. Available home ranges for each species were sampled within the area considered available to all individuals of that species. To define the boundaries for sampling available habitat, we generated 100% minimum convex polygons (MCPs) encompassing all GPS locations of all individuals for each species [79]. Using ArcGIS, we then created a buffer around all GPS locations for each species that was equal to the radius of the median circular seasonal home range (12,402 m for dingoes and 2811 m for feral cats) and merged this with the MCPs. We calculated the proportional availability of woodland for dingoes and grassland for feral cats in the used and randomly-sampled available home ranges, then used a resource selection function for each species based on logistic regression to compare used to available samples [78, 80]. We weighted available data to used data as 0.016:1 for dingoes (n_obs_ = 16) and 0.025:1 for cats (n_obs_ = 25), to allow for a balanced comparison and avoid inflating statistical precision [78].

### Fine-scale habitat selection

We examined fine-scale habitat selection (prediction 4 and 5) – i.e. third-order habitat selection – by dingoes and feral cats using separate step selection functions (SSFs) for each species [81]. SSFs link each observed GPS location to a set of random control locations or “steps” that are considered available but not selected by an individual animal at that time. We used Geospatial Modelling Environment (v. 0.7.2.1 Spatial Ecology LLC) to generate 20 random alternate locations for each observed GPS fix based on the empirical step length and turning angle distributions across all individuals for each species. We then attributed each observed and control location to the mapped habitat features using ArcGIS.

Habitat selection as a SSF was modelled as mixed effects Cox proportional hazard model for each species in R [82] using the ‘coxme’ package [83]. With Cox models, the selection coefficient for each habitat feature is estimated by conditional logistic regression and expressed as the log odds ratio of that feature being chosen relative to a reference habitat state. We specified one model for each species. We combined road and habitat type into one variable with four categories (i.e. on-road in open woodland, off-road in open woodland, on-road in grassland and off-road in grassland) with the reference community set as off-road in grassland; this allowed us to derive coefficients for each level, facilitating the interpretation of results. We also included a random intercept term for individual and a random coefficient for vegetation type. The inclusion of random intercepts in SFFs can be particularly beneficial when there is a discrepancy in sample size among individuals, as was the case here, while the random slope term for vegetation type allowed us to examine individual variation in responses to this covariate [84]. No pairs of the landscape features showed a Pearson’s correlation greater than 0.39 for either species; thus, all features were included in the models.

After fitting the models for each species, we examined deviance residuals for evidence of serial autocorrelation [85]. To do this, we fitted an intercept-only linear mixed-effects model to the residuals grouped by individual animal (i.e., a random intercept) for each species. Next, we plotted the autocorrelation function and determined the appropriate lag where autocorrelation became non-significant (α = 0.05). We then performed a sensitivity analysis by rerunning our original models with data grouped to these lags and examined the difference between model outputs of the sensitivity analysis and the original models.

We did not include time of day or sex as covariates in the models but rather examined the influence of these factors on habitat selection by subsetting the data and running the models for each subset group within each species separately. For time of day, we subset data as either diurnal or nocturnal for each species. We were only able to examine the effect of sex on habitat selection in dingoes, due to the small number of female feral cats sampled (*n* = 3).

## Results

Seventeen adult dingoes (6 ♂, 11 ♀) and 29 adult feral cats (24 ♂, 5 ♀) were captured between June 2013 and 2014 (Additional file 1: Table S1). One adult female cat was euthanized because of trap injuries. The remaining animals were fitted with GPS collars. Two collars representing one male and one female cat were not recovered, and one collar on a female dingo and one collar on a male cat had transmission errors and were discarded from analysis. As a result, pre-baiting data were collected from 16 dingoes (all in 2014) and 25 feral cats (six in 2013 and 19 in 2014). Following bait deployment, four of these dingoes stopped moving within five days, and 15 feral cats (four in 2013 and 11 in 2014) stopped moving within nine days – we suspect death from toxic bait ingestion.

Only data from the pre-baiting period each year were included in the analysis. This provided a total of 11,160 GPS fixes from 16 dingoes (range: 219–840, mean = 698 fixes per individual) and 5214 GPS fixes from 25 feral cats (range: 84–391, mean = 209 fixes per individual) after screening removed 13 dingo and 12 feral cat fixes. Total fixes represented between 14 and 70 days of location data for each animal. The average GPS-collar fix rate for these collars was 98.5% (range: 93.8–100%) for dingoes and 92.6% (range: 72.9–98.9%) for cats. Cut-off distances for movement (> 20 m for dingoes and > 35 m for feral cats) indicated that dingoes were moving 73.0% and cats 86.4% of the time. Fixes below the cut-off distances for movement were considered resting and removed. The remaining ‘moving’ fixes provided 8082 discrete habitat choices or ‘steps’ for dingoes and 4242 for feral cats (Additional file 1: Table S1). There was a slight female bias in the analysed dingo sample (6 ♂, 10 ♀) and a strong male bias in the cat sample (22 ♂, 3 ♀). The mean weight (± s.e.) of collared animals for this study was 16.34 ± 0.72 (range: 13.0–23.0) kg for dingoes and 4.21 ± 0.15 (range: 2.5–5.5) kg for feral cats.

### Home range size

**Fig. 2:**
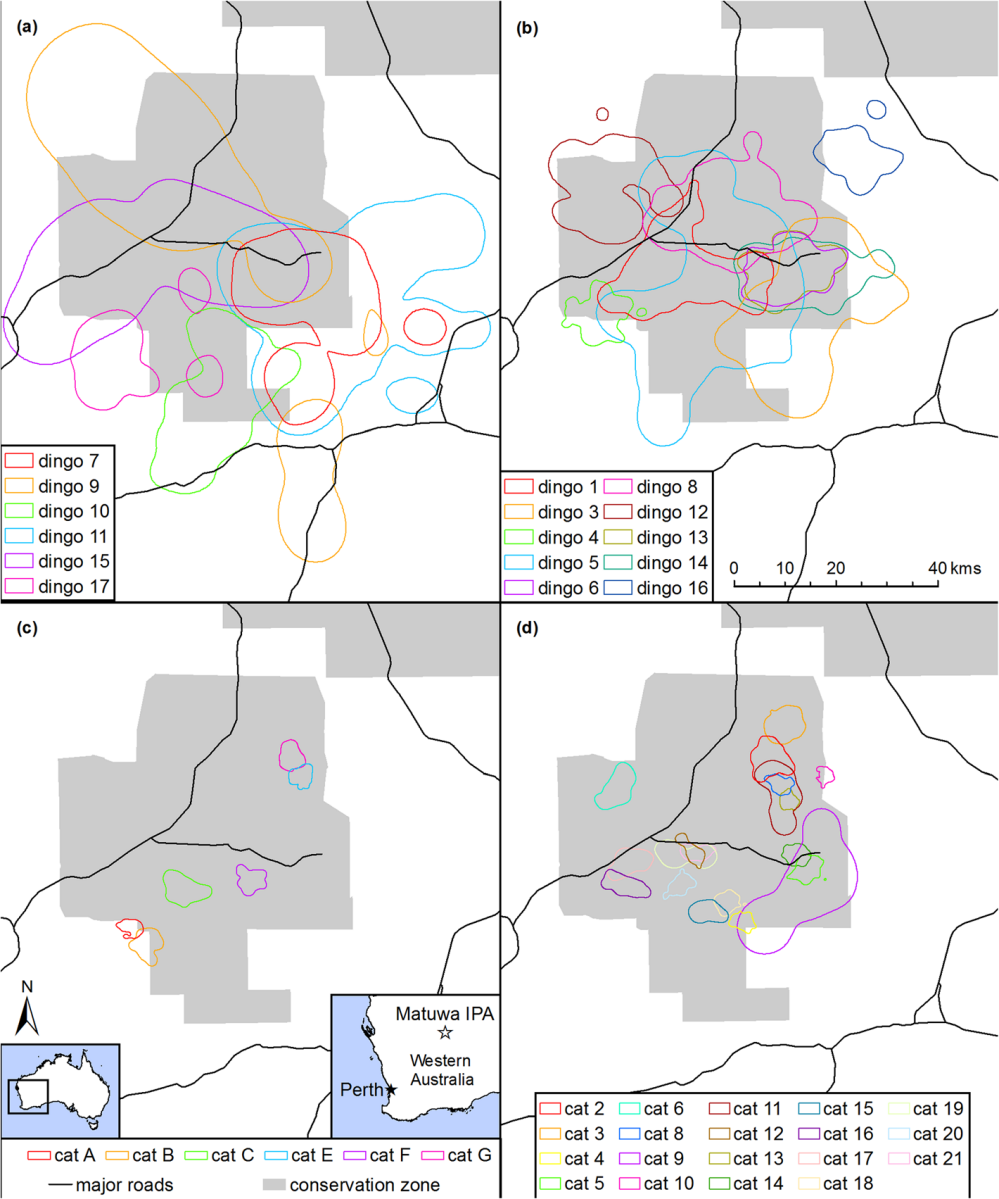
Utilization distributions (95% KDE) representing seasonal home ranges of male dingoes (**a**), female dingoes (**b**), feral cats captured in 2013 (**c**) and feral cats captured in 2014 (**d**) at Matuwa IPA and surrounding properties in semi-arid Western Australia

Kernel density estimates for all 16 dingoes and 25 feral cats reached asymptotes and were used in the analysis. Dingoes had substantially larger seasonal home ranges (95% kernel isopleth; β ± SE: 1.25 ± 0.11, t = 11.39, *p* < 0.001) and seasonal core home ranges (50% kernel isopleth; 1.14 ± 0.12, t = 9.45, *p* < 0.001) than feral cats (Table 1, Fig. 2, see also Additional files 2 (dingoes) and 3 (feral cats) for asymptote analyses and plots of 95 and 50% KDE utilisation distributions for each individual). There was some overlap in home range size, with the largest feral cat seasonal home range being larger than the five smallest dingo home ranges. However, the remaining feral cat seasonal home ranges were all smaller than all the smallest dingo home ranges. Male dingoes had substantially larger seasonal home ranges (0.43 ± 0.16, t = 2.601, *p* = 0.021) and seasonal core home ranges (11,733 ± 4898, t = 2.40, *p* = 0.031) than female dingoes (Table 1).

**Table 1:** Median, (IQR) and [min – max] of seasonal home range (95% KDE) and seasonal core home range (50% KDE) size of dingoes & feral cats at Matuwa IPA and surrounding properties, Western Australia

Species	n	95% KDE (ha)	50% KDE (ha)
Dingo	16	48,325 (24,220 - 115,703) [10,971 – 191,227]	11,133 (4732 - 24,630) [1250 - 36,561]
Male	6	101,834 (52,332 - 166,706) [33,594- 191,227]	22,496 (11,773 - 30,623) [3962 - 36,561]
Female	10	36,515 (18,943 - 58,773) [10,971 - 129,492]	7521 (4035-15,243) [1250 - 26,378]
Cat	25	2482 (1682 - 4434) [913–33,518]	591 (385–1221) [205–8645]
Male	22	2565 (1726 - 4429) [913–33,518]	618 (397–1348) (205–8645)
Female	3	1681 (1528 - 4858) [1528 - 4858]	410 (284–1099) [284–1099]

### Broad-scale habitat selection

The proportion of woodland within observed dingo seasonal home ranges varied from 51 to 100% (median 86%). There was no evidence that dingoes selectively located their seasonal home ranges in relation to the availability of woodland (β_woodland_ ± se: 0.47 ± 1.87, z = 0.251, *p* = 0.802; Additional file 1: Figure S1a). The proportion of grassland within observed cat seasonal home ranges varied from < 1 to 86% (median 43%). Overall, feral cats selected for home range locations that had a higher proportion of grassland than random (β_grassland_ ± se: 2.07 ± 0.95, z = 2.179, *p* = 0.029; Additional file 1: Figure S1b).

### Fine-scale habitat selection

**Fig. 3:**
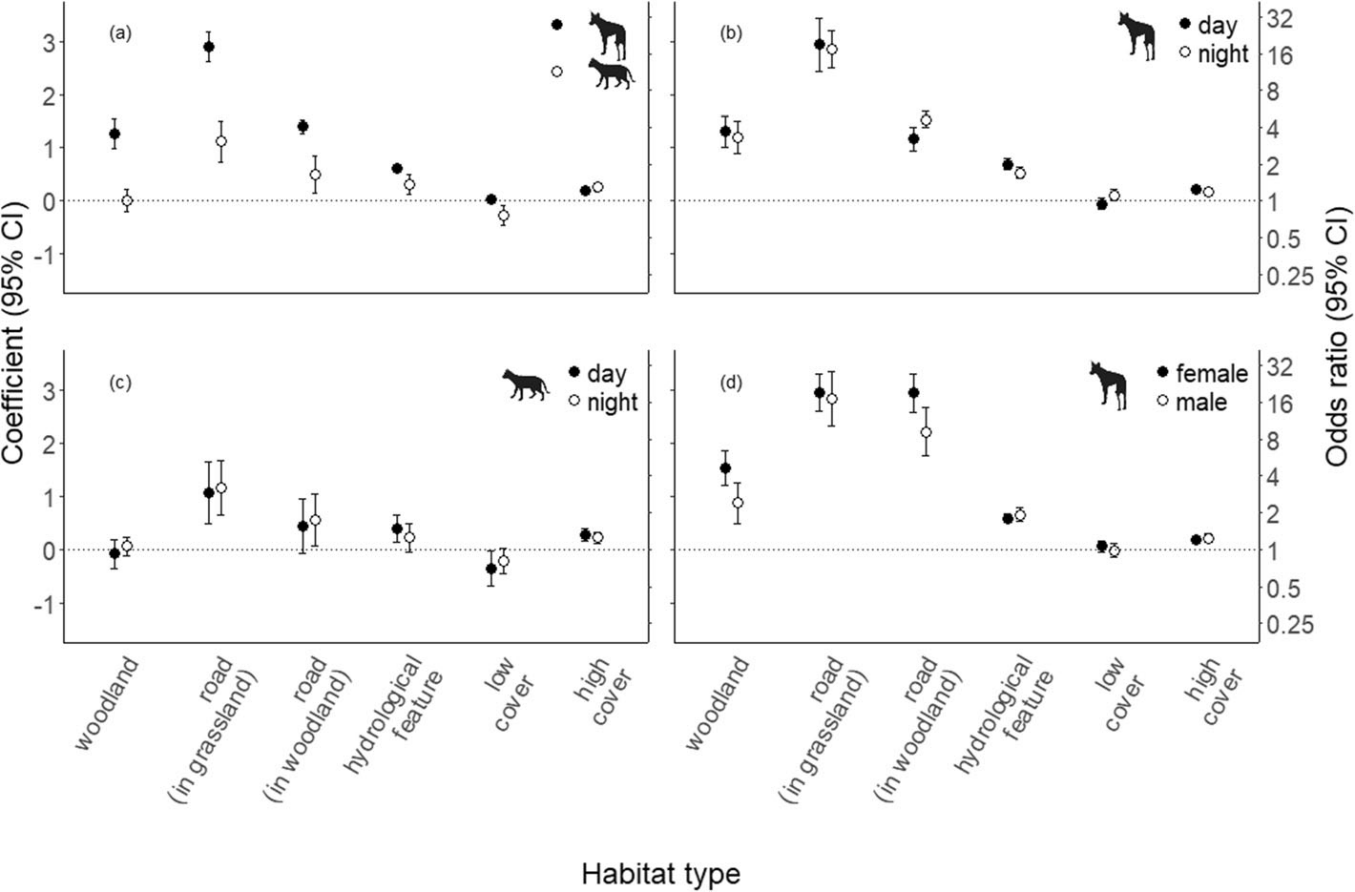
Regression coefficients of population-level (fixed) effects for habitat features included in mixed-effects step-selection function models comparing dingoes and feral cats (**a**) at Matuwa IPA and surrounding properties in semi-arid Western Australia. Model runs also included data subset by time of day (diurnal or nocturnal) for dingoes (**b**) and feral cats (**c**), and by sex for dingoes (**d**). 95% CIs above zero indicate significant selection for the habitat feature within the home range, those below indicate significant avoidance, and those overlapping zero indicate no significant selection of the feature relative to availability

In their fine-scale movements, dingoes selected strongly for open woodlands, whereas cats used open woodlands and grasslands in proportion to their availability within their home range (Fig. 3a, Additional file 1: Table S2). Both predators selected for roads in each vegetation type but selected more strongly for roads in grasslands than roads in open woodlands (relative to non-road habitats in the same vegetation type). The odds of dingoes selecting roads was 18.22 times higher in grasslands and 4.04 times higher in open woodlands than the odds of selecting non-road locations in those same vegetation types. For cats, the odds of selecting roads was 3.07 times higher in grasslands and 1.65 times higher in open woodlands compared to non-road locations in those same vegetation types. Both species also selected for hydrological features and high vegetation cover. Dingoes showed no response to low cover, whereas feral cats selected against areas with low cover. Our results also suggested that there was little difference between diurnal and nocturnal selection of habitat features by dingoes (Fig. 3b) or feral cats (Fig. 3c) although there were slight variations in selection patterns for male and female dingoes (Fig. 3d).

Results of the intercept-only linear mixed-effects models showed that autocorrelation was near zero after a lag of 5 observations (10 h) for dingoes and after 2 observations (8 h) for cats. Sensitivity tests of models including these lags suggested that autocorrelation impacts were minimal as only very minor changes in the model coefficients and standard errors were observed (Additional file 1: Table S3). Therefore, outputs for the full datasets are presented.

## Discussion

Understanding how apex- and meso-predators use landscapes in sympatry can inform relevant ecological theory such as species niches and competition, and in an applied context, help inform management decisions regarding pest control and wildlife conservation. We developed and tested predictions about the space use of a native apex predator (dingoes) and a sympatric, invasive mesopredator (feral cats) by examining their home ranges and habitat selection. Our predictions that dingoes would exhibit larger home ranges than feral cats and that male dingoes would have larger home ranges than females were supported by our results. Dingoes did not exhibit broad-scale habitat selection for open woodlands, but feral cats selected for grasslands at this scale. Our results also supported our prediction that dingoes would select for open woodlands, roads and hydrological features in their fine-scale movements. Contrary to our expectations, however, feral cats did not avoid these features. Instead, feral cats also selected for roads and hydrological features in their fine-scale movements but exhibited no third-order habitat selection for vegetation type. Our final prediction that dingoes would select for low vegetation cover and that feral cats would select for high vegetation cover was partially supported: both dingoes and feral cats selected for high vegetation cover, but feral cats also selected against low cover whereas dingoes did not. Our results suggest that mesopredators do not always avoid the same habitat features as sympatric apex predators but can coexist with them even at fine spatial scales. By understanding the habitat preferences of an invasive mesopredator in the presence of a native apex predator, our study emphasizes the importance of spatial considerations in vertebrate pest management.

The 20-fold difference in seasonal home range size observed between dingoes and feral cats was expected due to their difference in body size, energetic requirements and size of prey [16]. In arid regions of Australia, dingoes tend to prey on large macropodids (*Macropus* spp.) which often exceed their own weight [86, 87], while feral cats feed primarily on small mammals, birds and reptiles that are much smaller than their own body size [86, 88]. Dingo males also had larger seasonal home ranges than females. This difference is common among carnivores and is frequently associated with sexual dimorphism or the maximization of mating opportunities by males [52]. Dingo and feral cat home range estimates vary widely across Australia and between ecosystems [89–91]; however the seasonal home range estimates in this study are similar to other observations from arid regions of Australia [64, 92–94].

For each predator, we examined both broad-scale and fine-scale habitat selection across the two dominant vegetation types: open woodlands and grasslands. We found evidence to support broad-scale habitat selection of grasslands by feral cats but no evidence of habitat selection of woodlands by dingoes at this scale. In contrast, dingoes selected for open woodlands in their fine-scale movements, whereas third-order habitat selection for vegetation type was equivocal among feral cats; although some individual cats likely selected either for or against open woodlands. Strong selection for open woodlands by dingoes within their home ranges was most likely related to the presence of their main prey, macropodids [86], which typically prefer open woodlands to hummock grasslands in semi-arid ecosystems [53].

Second-order habitat selection for grasslands by feral cats suggests that they prefer to establish home ranges in this vegetation type even though they may not specifically select for it in their fine-scale movements. It is likely that feral cat selection at this scale is driven by prey availability. Hummock grasslands in our study site, and in semi-arid ecosystems more generally, support more abundant and diverse small vertebrates – which make up the bulk of feral cat diets – than open woodlands [54, 95, 96]. At the same time, feral cat second-order habitat selection for grasslands could be influenced by avoidance of dingoes. Although the two predators co-occur at fine spatial scales, feral cats are known to avoid dingoes temporally or spatially [89], similar to other sympatric apex predators and mesopredators [97–100]. To understand which of these factors is important for feral cat habitat selection, we recommend studies which manipulate either resource availability (e.g. via fire) or dingo abundance (e.g. via exclusion fencing or using existing contrasts in poison baiting) within both habitat types.

Dingoes selected very strongly for roads in both habitats in their fine-scale movements. This finding is consistent with a large body of evidence that demonstrates that many apex predators including wolves [34, 101], bears [27] and African wild dogs [102] select for roads and other linear features especially where traffic or human presence is low. In Australia, Robley, Gormley [55] documented selection for roads by dingoes in a forested region of eastern Victoria, and it is generally accepted that roads provide important movement corridors for dingoes [50]. However, our results indicate a much stronger selection for roads in grasslands than in open woodlands. This stronger selection for roads in grasslands may reflect the difference in utility of these two habitat types. With reduced food resources in grasslands, dingoes may primarily use roads in this habitat to travel efficiently to more productive (i.e. open woodland) foraging areas of their home ranges. However, it is also possible that dingoes select roads more strongly in grasslands to avoid traversing through spiky grasses.

Contrary to our predictions, feral cats selected for roads in both vegetation types, despite dingoes also strongly selecting for this feature. The use and selection of roads by feral cats has been well-documented globally and within Australia [103]. Feral cat selection for roads was substantially weaker than that of dingoes which may be a function of the smaller size of feral cat home ranges and hence the lower likelihood of encountering roads relative to their density in the study area. Like dingoes, feral cats selected for roads more strongly in grasslands than in open woodlands. However, unlike dingoes, feral cats also showed second-order habitat selection for grasslands. The stronger selection for roads in grasslands may be because the higher understory cover and high density of spiky spinifex grasses in grasslands present more resistance to movement than open woodlands. It is also possible that feral cats may use roads less in open woodlands to limit their exposure to dingoes where encounters are more likely. Without manipulating resource or dingo abundance, it is difficult to determine which mechanism is driving habitat choice — these possibilities require further investigation.

As predicted, dingoes selected for hydrological features within their home ranges. Selection for these features is likely due to higher prey availability and the presence of water, and/or the use of waterways as movement corridors, as has been documented for other predators [104–106]. Thomson [57] also found that dingoes selected for riparian areas and proposed that the location of water was an important factor in determining the distributions of both dingoes and their prey, while Harden [56] found that dingoes frequently travelled along streams during the course of their regular movements.

In contrast to our prediction, we found that feral cats also selected for hydrological features. While this has previously been documented in other parts of Australia [107, 108], to our knowledge this study represents the first evidence of this preference in arid regions of the country. Unlike dingoes [109], feral cats do not need to drink regularly [110], hence their preference for hydrological features is probably related to the presence of prey, physiological constraints (a cooler, thermal refuge), and/or use as movement corridors.

Our results did not support our prediction that dingoes would preferentially select low vegetation cover; rather they selected for high vegetation cover. Dingo selection for high vegetation cover may be related to the presence of at least one of their main prey species, the common wallaroo (*Macropus robustus*), which typically prefers habitat with moderate cover and complexity [111]. It may also reflect the dingoes’ need for concealment and shade in hot arid climates [50].

Like dingoes, feral cats also selected for areas of high vegetation cover but unlike dingoes they simultaneously selected against areas of low vegetation cover. Many felids prefer areas of high vegetation cover for stalking and ambushing prey; feral cats have been documented using high vegetation cover both globally [103] and within Australia [93, 94, 112]. Avoidance of low vegetation cover is probably important for shelter and concealment from other predators, such as dingoes. However, McGregor, Legge [108] found that cats select more strongly for the edges of open habitats, particularly newly-burnt areas and concluded that this was probably to maximize hunting efficiency. We did not investigate predator habitat selection with respect to fire attributes but acknowledge that this is likely to be of value particularly for fire-prone landscapes [113], and it should be a focus for further work in our study region.

## Conclusions and management implications

Roads and other linear features have disproportionately large effects on ecological processes, despite occupying a relatively small proportion of land [114, 115]. Previous studies have quantified predator selection between different types of linear features such as between trail [116] or road types [35], but to our knowledge this is the first study to quantify the strength of selection by multiple predator species for roads or linear features across different habitat types. We found that both predators selected more strongly for roads within grasslands than roads within open woodlands. Because many predators – particularly carnivores – are known to use roads, studies that investigate the variability in the selection for roads across different habitats have the potential to greatly improve our understanding of predator space use and help inform predator management.

Our case study highlights the potential for strategically designed and spatially-heterogenous pest control programs to limit non-target impacts and maximise conservation outcomes. We found that both dingoes and feral cats selected for hydrological features, high vegetation cover and roads. Therefore, invasive predator control in these parts of the landscape should focus on protecting key sites of high biodiversity value (such as lakes or waterholes) by employing techniques that are specific to feral cats such as trapping or shooting at scales that are feasible for management. Aerial dispersal of predator baits which do not discriminate between feral cats and dingoes is currently applied once a year over the entire study site. Restricting baiting to grasslands away from roads could increase the specificity of this management and reduce non-target impacts on dingoes, as feral cats showed some preference for grasslands while dingoes selected strongly for open woodlands and occurred very infrequently off-road in grasslands.

## Supplementary information

**Additional file 1: Table S1.** Details of animal capture and GPS-collar data collection for dingoes and feral cats at Matuwa Indigenous Protected Area, Western Australia. **Table S2.** Parameter estimates and odds-ratios for mixed-effects step-selection function models of dingo and feral cat habitat selection at Matuwa Indigenous Protected Area and surrounding properties in semi-arid Western Australia. **Table S3.** Results of sensitivity tests with data grouped by serial autocorrelated lags. **Figure S1.** Logistic regressions of proportional availability of woodland and grassland in the used and randomly-sampled available home ranges for dingoes and feral cats.

**Additional file 2.** Asymptote analysis and KDE utilisation distributions for individual dingo home ranges.

**Additional file 3.** Asymptote analysis and KDE utilization distributions for individual feral cat home ranges.

## Data Availability

The datasets used and/or analysed during the current study are available from the corresponding author on reasonable request.

## Abbreviations

GPS: Global Positioning System
IPA: Indigenous Protected Area
KDE: Kernel Density Estimation
MCP: Minimum Convex Polygon
SSF: Step Selection Function
UD: Utilisation Distribution
