# Supplementary material for: Space use and habitat selection of an invasive mesopredator and sympatric, native apex predator
Source: Mov Ecol. 2020 May 4;8:18. doi: 10.1186/s40462-020-00203-z (PMC7197163; doi:10.1186/s40462-020-00203-z)
Supplement: Supplementary file 1 — Additional file 1: Table S1. Details of animal capture and GPS-collar data collection for dingoes and feral cats at Matuwa Indigenous Protected Area, Western Australia. Table S2. Parameter estimates and odds-ratios for mixed-effects step-selection function models of dingo and feral cat habitat selection at Matuwa Indigenous Protected Area and surrounding properties in semi-arid Western Australia. Table S3. Results of sensitivity tests with data grouped by serial autocorrelated lags. Figure S1. Logistic regressions of proportional availability of woodland and grassland in the used and randomly-sampled available home ranges for dingoes and feral cats. [file 40462_2020_203_MOESM1_ESM.docx]

**Additional file 1.**

**Table S1.** Details of animal capture and GPS-collar data collection for dingoes and feral cats at Matuwa IPA, Western Australia. Seasonal home ranges were calculated at the 95% and 50% KDE utilization distribution (UD) using the reference bandwidth; the percentage of each UD and the percentage of total fixes occurring in grassland habitats is also listed. Animals for which no data was collected were the result of collar failure or collar drop-off failure‡; or poor collar fix rate ⸸. One additional female cat not listed here was euthanised due to trap injuries.

| **species** | **name** | **sex** | **mass (kg)** | **date collared** | **dates of data collection** | **no. of days** | **fix interval: % success (n successful)** | **no. of steps** | **95% KDE (ha)** | **%KDE in grassland** | **50% KDE (ha)** | **% core KDE in grassland** | **% fixes in grassland** |
| --- | --- | --- | --- | --- | --- | --- | --- | --- | --- | --- | --- | --- | --- |
| cat | cat 1 | F | 3.9 | 25/04/14 | ‡ | ‡ | ‡ | ‡ | ‡ | ‡ | ‡ | ‡ | ‡ |
|  | cat 2 | M | 4.5 | 27/04/14 | May 1 - Jul 9 | 70 | 4 hrs: 92% (391) | 339 | 5198 | 3.9 | 1582 | 4.7 | 4.6 |
|  | cat 3 | M | 4.1 | 28/04/14 | May 1 - Jul 9 | 70 | 4 hrs: 88% (370) | 310 | 4747 | 2.4 | 1403 | 7.7 | 4.1 |
|  | cat 4 | M | 5.0 | 04/05/14 | May 5 - Jul 9 | 66 | 4 hrs: 73% (289) | 195 | 1496 | 22.5 | 353 | 0.0 | 8.0 |
|  | cat 5 | M | 4.5 | 08/05/14 | May 9 - Jul 9 | 63 | 4 hrs: 93% (347) | 293 | 2647 | 0.7 | 587 | 0.0 | 1.2 |
|  | cat 6 | M | 3.5 | 12/05/14 | May 13 - Jul 9 | 58 | 4 hrs: 95% (330) | 287 | 4444 | 84.0 | 1342 | 93.5 | 86.4 |
|  | cat 7 | M | 4.5 | 05/06/14 | Jun 6- Jul 9 | 33 | 4 hrs: 44% (84) | ⸸ | ⸸ | ⸸ | ⸸ | ⸸ | ⸸ |
|  | cat 8 | F | 2.5 | 06/06/14 | Jun 7 - Jul 9 | 33 | 4 hrs: 91% (180) | 133 | 1528 | 8.4 | 410 | 3.8 | 8.3 |
|  | cat 9 | M | 3.0 | 07/06/14 | Jun 8 - Jul 9 | 32 | 4 hrs: 77% (146) | 96 | 33518 | 17.1 | 8645 | 24.1 | 35.6 |
|  | cat 10 | M | 4.9 | 07/06/14 | Jun 8 - Jul 9 | 32 | 4 hrs: 96% (184) | 152 | 913 | 74.2 | 205 | 86.5 | 82.6 |
|  | cat 11 | M | 5.0 | 08/06/14 | Jun 9 - Jul 9 | 31 | 4 hrs: 99% (184) | 169 | 8044 | 4.7 | 2139 | 3.7 | 1.6 |
|  | cat 12 | F | 3.7 | 08/06/14 | Jun 9 - Jul 9 | 31 | 4 hrs: 88% (164) | 115 | 1681 | 80.1 | 284 | 85.6 | 75.0 |
|  | cat 13 | M | 3.9 | 09/06/14 | Jun 10 - Jul 9 | 30 | 4 hrs: 94% (169) | 150 | 1157 | 1.3 | 315 | 0.0 | 3.6 |
|  | cat 14 | M | 5.1 | 12/06/14 | Jun 13 - Jul 9 | 27 | 4 hrs: 94% (152) | 135 | 1911 | 0.1 | 493 | 0.0 | 0.7 |
|  | cat 15 | M | 4.6 | 13/06/14 | Jun 14 - Jul 9 | 26 | 4 hrs: 98% (153) | 139 | 2658 | 42.2 | 872 | 39.4 | 39.9 |
|  | cat 16 | M | 5.1 | 13/06/14 | Jun 14 - Jul 9 | 26 | 4 hrs: 96% (150) | 136 | 3262 | 57.6 | 762 | 59.9 | 62.0 |
|  | cat 17 | M | 4.5 | 13/06/14 | Jun 14 - Jul 9 | 26 | 4 hrs: 90% (139) | 114 | 3764 | 46.1 | 1088 | 43.5 | 17.3 |
|  | cat 18 | M | 3.9 | 14/06/14 | Jun 15 - Jul 9 | 25 | 4 hrs: 95% (142) | 113 | 1814 | 75.8 | 536 | 78.3 | 64.8 |
|  | cat 19 | F | 3.0 | 14/06/14 | Jun 15 - Jul 9 | 25 | 4 hrs: 99% (148) | 114 | 4858 | 64.1 | 1099 | 70.6 | 86.5 |
|  | cat 20 | M | 4.0 | 14/06/14 | Jun 15 - Jul 9 | 25 | 4 hrs: 99% (148) | 139 | 1852 | 79.5 | 408 | 89.9 | 83.8 |
|  | cat 21 | M | 4.5 | 15/06/14 | Jun 16 - Jul 9 | 24 | 4 hrs: 99% (142) | 126 | 1682 | 85.6 | 451 | 73.5 | 71.8 |
|  | cat A | M | 5.5 | 13/06/13 | Jun 14 - Jul 5 | 22 | 2 hrs: 98% (264) | 181 | 1388 | 71.1 | 315 | 60.5 | 59.5 |
|  | cat B | M | 3.5 | 14/06/13 | Jun 15 - Jul 5 | 21 | 2 hrs: 98% (253) | 215 | 3199 | 80.6 | 902 | 90.2 | 75.1 |
|  | cat C | M | 4.2 | 16/06/13 | Jun 17 - Jul 5 | 19 | 2 hrs: 93% (218) | 159 | 4424 | 76.4 | 1364 | 90.1 | 81.2 |
|  | cat D | M | 4.7 | 18/06/13 | ‡ | ‡ | ‡ | ‡ | ‡ | ‡ | ‡ | ‡ | ‡ |
|  | cat E | M | 4.0 | 21/06/13 | Jun 22 - Jul 5 | 14 | 2 hrs: 92% (159) | 135 | 1741 | 9.1 | 362 | 6.5 | 3.1 |
|  | cat F | M | 4.5 | 20/06/13 | Jun 21 - Jul 5 | 15 | 2 hrs: 87% (163) | 111 | 2482 | 3.1 | 591 | 1.7 | 1.2 |
|  | cat G | M | 4.2 | 15/06/13 | Jun 16 - Jul 5 | 20 | 2 hrs: 93% (229) | 186 | 2300 | 2.0 | 645 | 0.6 | 0.4 |
| dingo | dingo 1 | F | 14.6 | 04/10/13 | May 1- Jul 4 | 65 | 2 hrs: 95% (772) | 562 | 44557 | 41.4 | 8403 | 31.6 | 9.2 |
|  | dingo 2 | F | 11.7 | 05/10/13 | ‡ | ‡ | ‡ | ‡ | ‡ | ‡ | ‡ | ‡ | ‡ |
|  | dingo 3 | F | 14.5 | 15/10/13 | May 1- Jul 9 | 70 | 2 hrs: 94% (838) | 597 | 78813 | 8.5 | 19387 | 6.2 | 0.8 |
|  | dingo 4 | F | 13.8 | 20/10/13 | May 1- Jul 9 | 70 | 2 hrs: 98% (837) | 540 | 10971 | 41.5 | 1250 | 26.3 | 14.8 |
|  | dingo 5 | F | 15.6 | 22/10/13 | May 1- Jul 9 | 70 | 2 hrs: 99% (840) | 617 | 129492 | 33.1 | 26378 | 16.4 | 3.5 |
|  | dingo 6 | F | 14.3 | 30/04/14 | May 1- Jul 9 | 70 | 2 hrs: 99% (834) | 631 | 20261 | 0.5 | 4500 | 0.0 | 0.4 |
|  | dingo 7 | M | 21.0 | 01/05/14 | May 2- Jul 9 | 69 | 2 hrs: 99% (822) | 601 | 75669 | 3.1 | 17294 | 1.2 | 0.1 |
|  | dingo 8 | F | 16.2 | 03/05/14 | May 4- Jul 9 | 67 | 2 hrs: 99% (792) | 582 | 52093 | 13.8 | 13862 | 0.1 | 3.5 |
|  | dingo 9 | M | 18.0 | 04/05/14 | May 5- Jul 9 | 66 | 2 hrs: 99% (785) | 579 | 191227 | 13.3 | 27697 | 10.8 | 3.3 |
|  | dingo 10 | M | 23.0 | 05/05/14 | May 6- Jul 9 | 65 | 2 hrs: 99% (771) | 552 | 58578 | 40.2 | 14377 | 19.6 | 13.5 |
|  | dingo 11 | M | 19.5 | 06/05/14 | May 7- Jul 9 | 64 | 2 hrs: 99% (761) | 559 | 158532 | 4.7 | 36561 | 0.3 | 0.3 |
|  | dingo 12 | F | 15.4 | 06/05/14 | May 7- Jul 9 | 64 | 2 hrs: 100% (767) | 526 | 39650 | 49.2 | 7769 | 35.7 | 4.0 |
|  | dingo 13 | F | 13.2 | 07/05/14 | May 8- Jul 9 | 63 | 2 hrs: 99% (749) | 557 | 21167 | 0.4 | 5427 | 0.2 | 0.1 |
|  | dingo 14 | F | 14.8 | 15/05/14 | May 16- Jul 9 | 55 | 2 hrs: 99% (655) | 508 | 33380 | 0.7 | 7273 | 0.1 | 0.2 |
|  | dingo 15 | M | 16.0 | 20/05/14 | May 21- Jun 8 | 18 | 2 hrs: 100% (219) | 160 | 127999 | 36.3 | 28643 | 52.1 | 16.0 |
|  | dingo 16 | F | 13.0 | 08/06/14 | Jun 9 - Jul 9 | 31 | 2 hrs: 99% (369) | 272 | 14989 | 0.0 | 2640 | 0.0 | 0.0 |
|  | dingo 17 | M | 18.5 | 09/06/14 | Jun 10 - Jul 9 | 30 | 2 hrs: 98% (349) | 240 | 33594 | 43.8 | 3962 | 54.7 | 22.6 |

**Table S2**. Parameter estimates and odds-ratios for mixed-effects step-selection function models of dingo and feral cat habitat selection at Matuwa IPA and surrounding properties in semi-arid Western Australia. Binary variables for open woodland and road were combined into one variable with 4 categories in order to derive coefficients for their interaction. The reference community was grassland/off-road except as indicated with (ǂ) where the reference community was to open woodland/off-road. This was done to facilitate a comparison of road vs off-road selection in woodland. Subscripts for road indicate vegetation type where selection occurs: reference vegetation type. Hydrological feature is populated with dummy variables and vegetation cover represents 3 classes with reference set to moderate cover.

| **Species** | **Fixed effects** | **Odds ratio** | **β** | **SE** | **Z** | **Pr(>\|z\|)** |  |
| --- | --- | --- | --- | --- | --- | --- | --- |
| dingo | open woodland | 3.53 | 1.26 | 0.14 | 8.84 | <0.0001 | *** |
|  | hydrological feature | 1.83 | 0.61 | 0.04 | 16.70 | <0.0001 | *** |
|  | low cover | 1.03 | 0.02 | 0.04 | 0.65 | 0.510 |  |
|  | high cover | 1.22 | 0.20 | 0.02 | 7.97 | <0.0001 | *** |
|  | road_grass:grass_ | 18.22 | 2.90 | 0.15 | 19.93 | <0.0001 | *** |
|  | road_wood:grass_ | 14.26 | 2.66 | 0.16 | 17.06 | <0.0001 | *** |
|  | road_wood:wood_ ǂ | 4.04 | 1.40 | 0.07 | 21.48 | <0.0001 | *** |
|  | **Random effects** | **Variance** |  |  |  |  |  |
|  | intercept | 0.72 |  |  |  |  |  |
|  | β (open woodland) | 0.19 |  |  |  |  |  |
| feral cat | open woodland | 1.01 | 0.01 | 0.11 | 0.07 | 0.95 |  |
|  | hydrological feature | 1.36 | 0.31 | 0.09 | 3.23 | 0.0012 | ** |
|  | low cover | 0.76 | -0.28 | 0.10 | -2.79 | 0.0052 | ** |
|  | high cover | 1.29 | 0.25 | 0.04 | 6.59 | <0.0001 | *** |
|  | road_grass:grass_ | 3.07 | 1.12 | 0.20 | 5.73 | <0.0001 | *** |
|  | road_wood:grass_ | 1.66 | 0.51 | 0.21 | 2.45 | 0.014 | * |
|  | road_wood:wood_ ǂ | 1.65 | 0.50 | 0.18 | 2.80 | 0.0052 | ** |
|  | **Random effects** | **Variance** |  |  |  |  |  |
|  | intercept | 0.56 |  |  |  |  |  |
|  | β (open woodland) | 0.21 |  |  |  |  |  |

**Table S3.** Results of sensitivity tests with data grouped by serial autocorrelated lags. Plots of the autocorrelation functions of fitted mixed-effects step-selection function models revealed that autocorrelation declined to near aero after a lag of 5 observations (10 hours) for dingoes and 2 observations (8 hours) for feral cats. Data was then regrouped by these lags and run again for each mixed-effects step-selection function model of each species as described in Table S1. Model outputs of these sensitivity analyses deviate only slightly from original models in Figure 3 (and see also Table S2) hence we concluded that autocorrelation was not a serious issue and that original model results were adequate.

| **Species** | **Fixed effects** | **Odds ratio** | **β** | **SE** | **Z** | **Pr(>\|z\|)** |  |
| --- | --- | --- | --- | --- | --- | --- | --- |
| dingo | open woodland | 3.53 | 1.26 | 0.14 | 8.88 | <0.0001 | *** |
|  | hydrological feature | 1.84 | 0.61 | 0.04 | 16.77 | <0.0001 | *** |
|  | low cover | 1.03 | 0.02 | 0.04 | 0.65 | 0.520 |  |
|  | high cover | 1.22 | 0.20 | 0.02 | 7.96 | <0.0001 | *** |
|  | road_grass:grass_ | 18.29 | 2.91 | 0.15 | 19.96 | <0.0001 | *** |
|  | road_wood:grass_ | 14.30 | 2.66 | 0.16 | 17.14 | <0.0001 | *** |
|  | road_wood:wood_ ǂ | 4.05 | 1.40 | 0.07 | 21.52 | <0.0001 | *** |
|  | **Random effects** | **Variance** |  |  |  |  |  |
|  | intercept | 0.70 |  |  |  |  |  |
|  | β (open woodland) | 0.19 |  |  |  |  |  |
| feral cat | open woodland | 1.01 | 0.01 | 0.11 | 0.07 | 0.95 |  |
|  | hydrological feature | 1.36 | 0.31 | 0.09 | 3.23 | 0.0012 | ** |
|  | low cover | 0.76 | -0.27 | 0.10 | -2.78 | 0.0054 | ** |
|  | high cover | 1.29 | 0.25 | 0.04 | 6.60 | <0.0001 | *** |
|  | road_grass:grass_ | 3.08 | 1.12 | 0.20 | 5.74 | <0.0001 | *** |
|  | road_wood:grass_ | 1.66 | 0.51 | 0.21 | 2.45 | 0.014 | * |
|  | road_wood:wood_ ǂ | 1.65 | 0.50 | 0.18 | 2.80 | 0.0052 | ** |
|  | **Random effects** | **Variance** |  |  |  |  |  |
|  | intercept | 0.56 |  |  |  |  |  |
|  | β (open woodland) | 0.21 |  |  |  |  |  |

|  |
| --- |

| 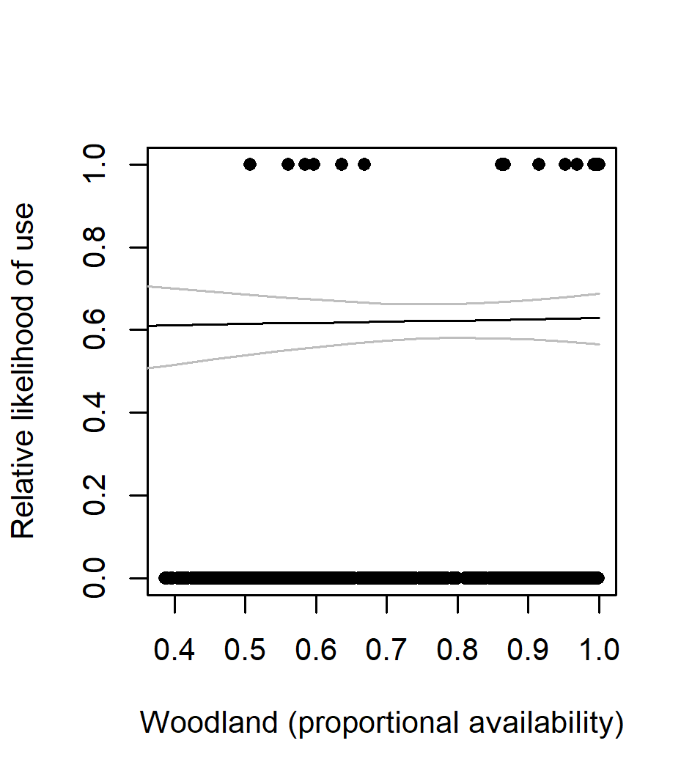  a) | 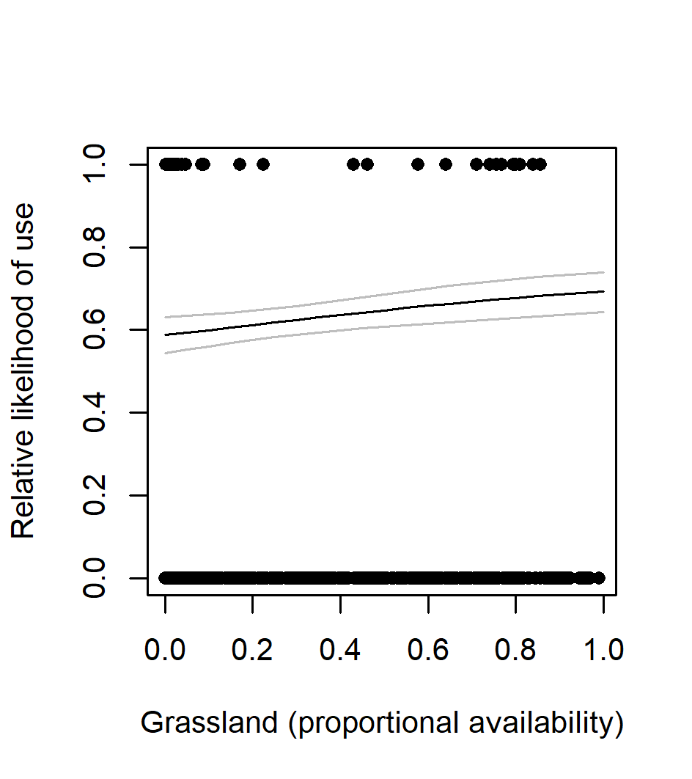  b) |
| --- | --- |

**Figure S1.** Logistic regressions of proportional availability of woodland and grassland in the used and randomly-sampled available home ranges for a) dingoes and b)feral cats.
