# Supplementary material for: Space use and habitat selection of an invasive mesopredator and sympatric, native apex predator
Source: Mov Ecol. 2020 May 4;8:18. doi: 10.1186/s40462-020-00203-z (PMC7197163; doi:10.1186/s40462-020-00203-z)
Supplement: Supplementary file 2 — Additional file 2. Asymptote analysis and KDE utilisation distributions for individual dingo home ranges. [file 40462_2020_203_MOESM2_ESM.docx]

**Additional file 2**. Asymptote analysis (left) and KDE utilisation distributions (right) for sixteen dingo home ranges. Asymptote data added randomly at 25-fix intervals for dingo seasonal home ranges calculated at the 95% KDE utilization distribution with number of fixes listed on the x-axis and area (m^2^) on the y-axis. Seasonal home ranges are sufficiently described when 75-100% of fixes are within 5% total kernal area (represented in grey) which was achieved for all individuals in the analysis. KDE utilisation distributions use the reference bandwidth showing 95% and 50% (core) isopleths; axes indicate UTM position coordinates in WGS 1984 zone 51S. Animals lised in sequential order from Table S1 starting with Dingo 1. (Animals with insufficient data are not represented).

| 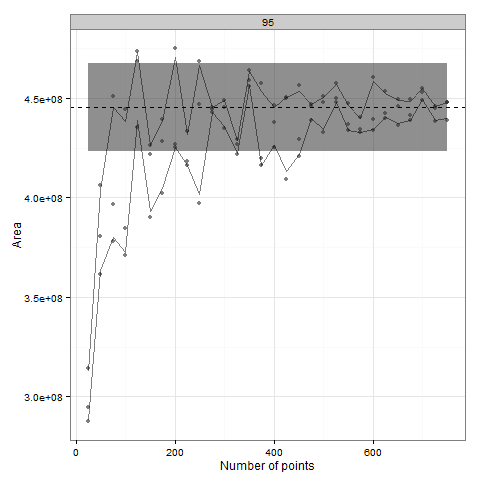 | 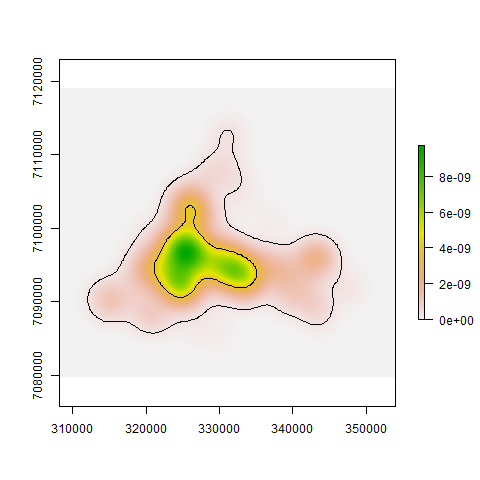 |
| --- | --- |
| 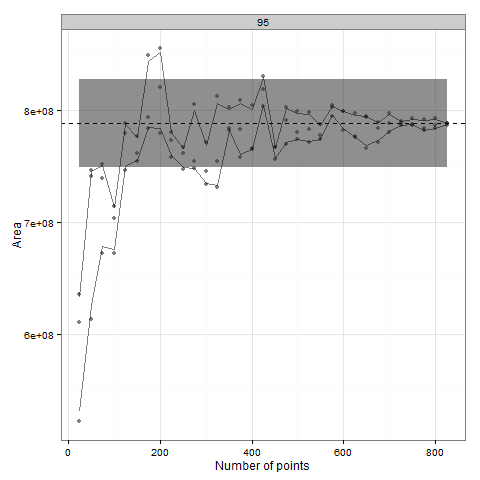 | 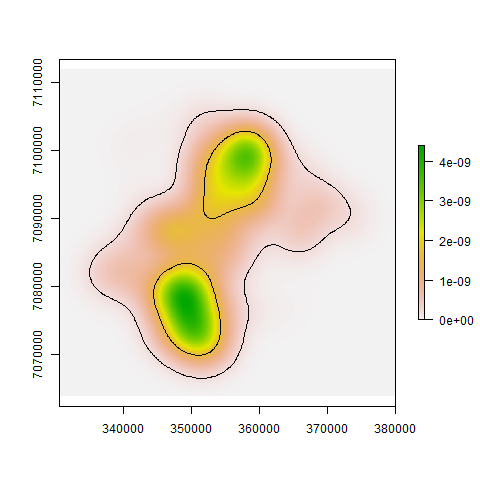 |
| 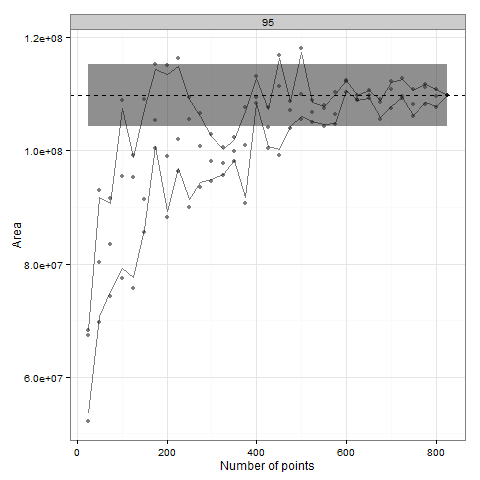 | 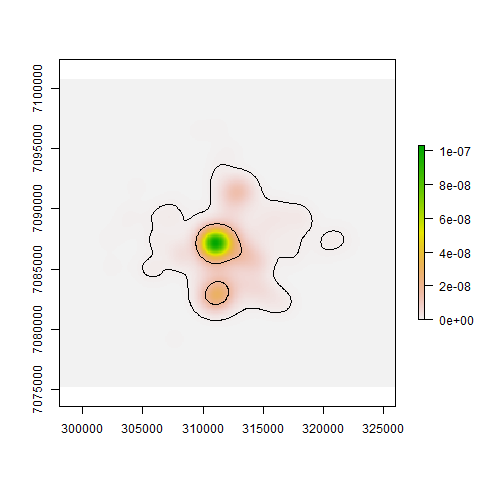 |
| 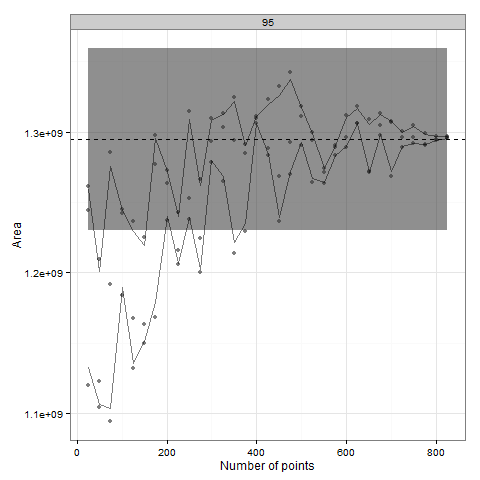 | 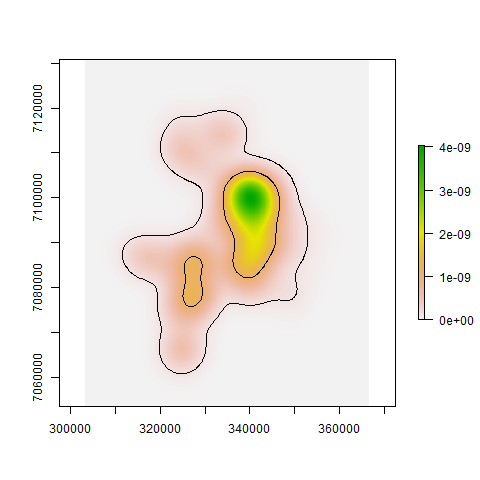 |
| 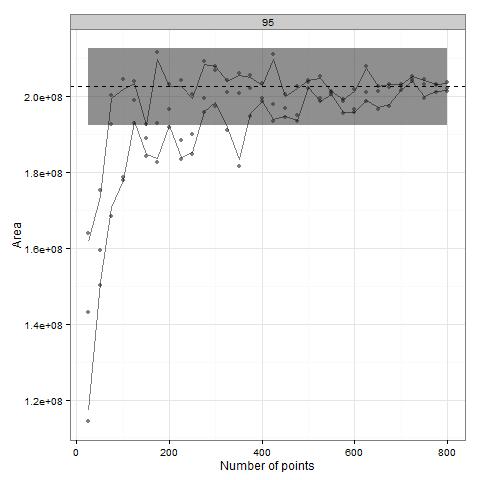 | 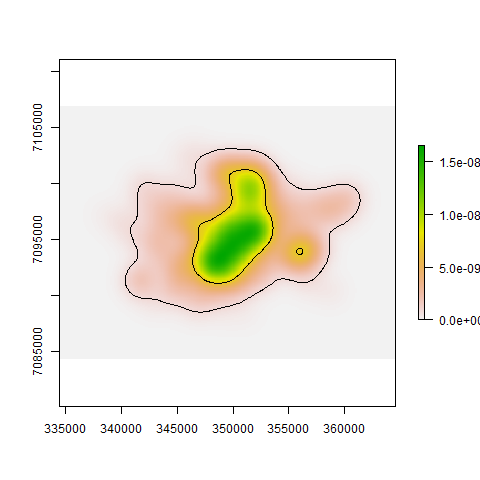 |
| 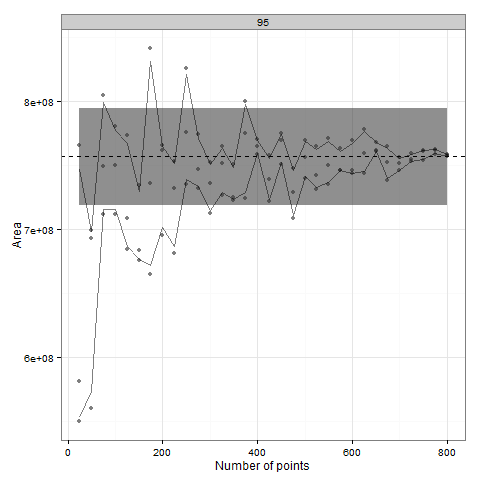 | 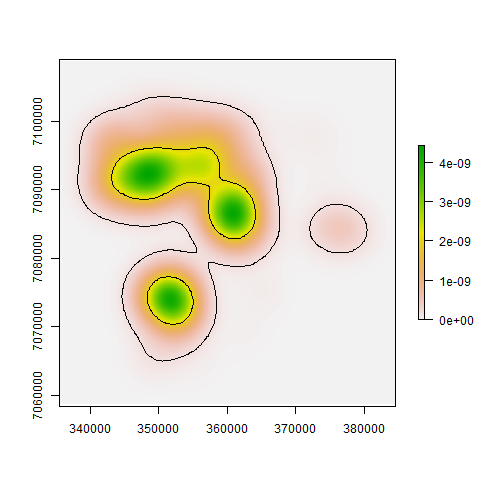 |
| 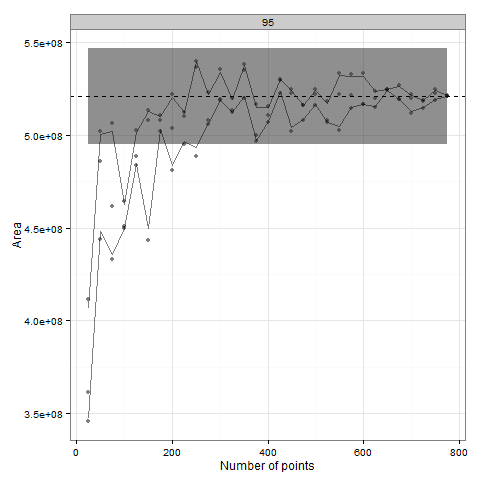 | 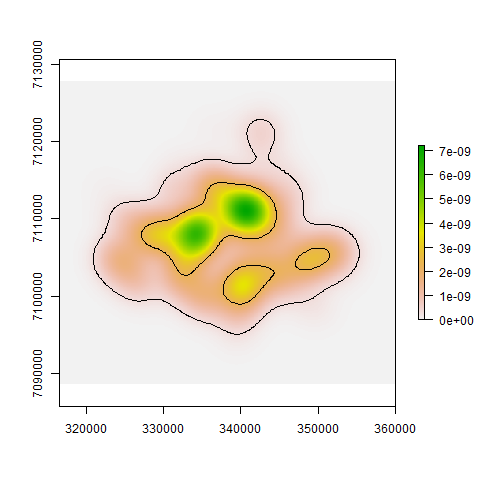 |
| 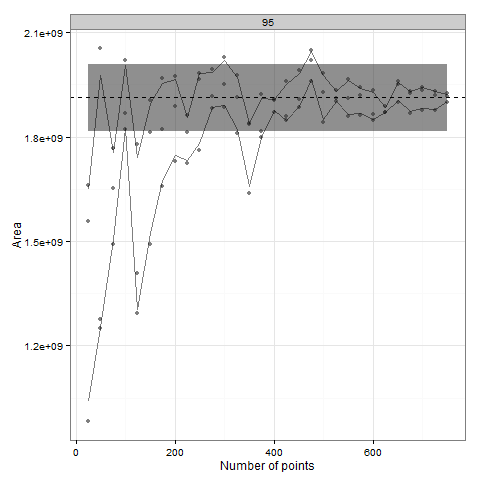 | 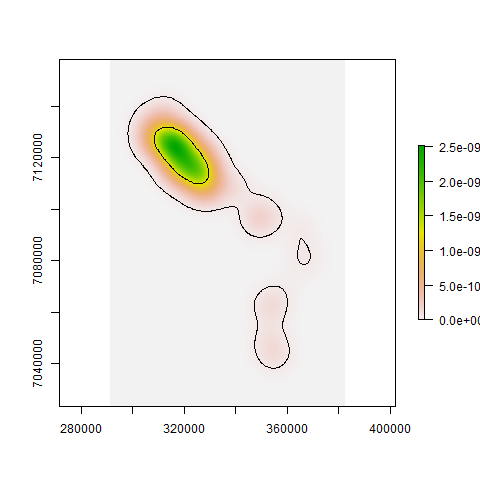 |
| 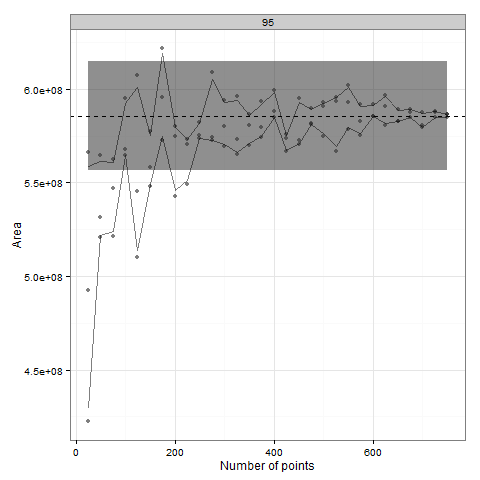 | 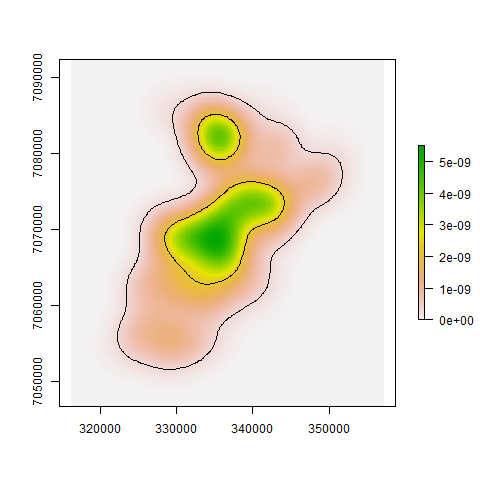 |
| 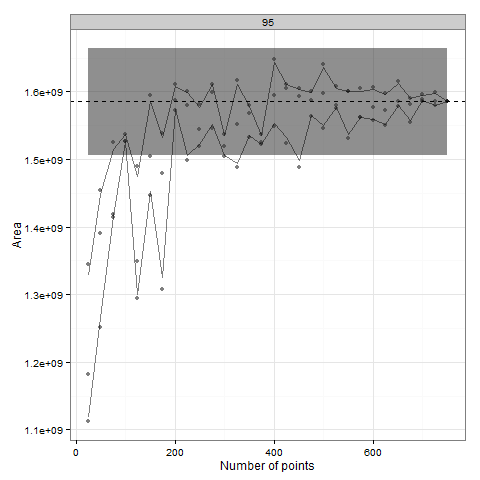 | 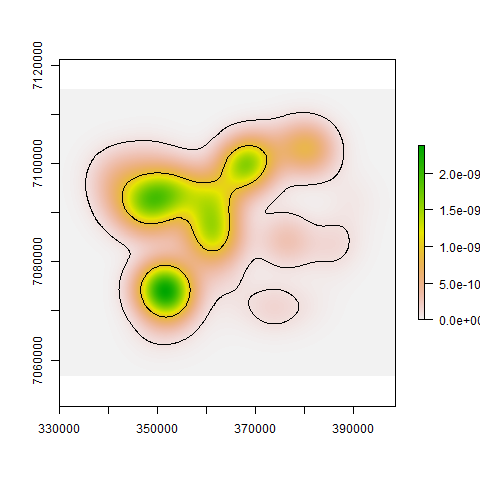 |
| 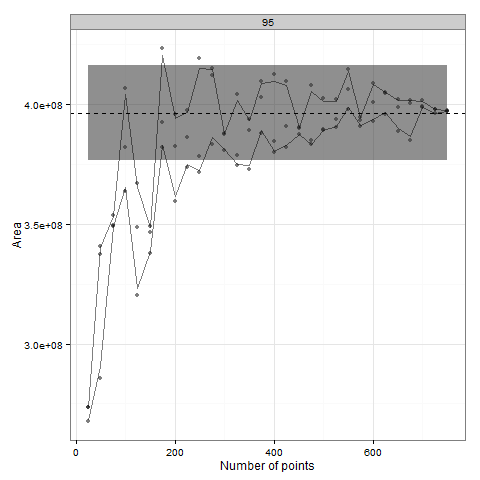 | 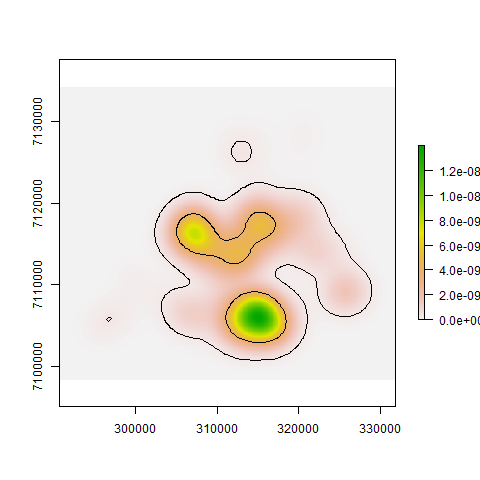 |
| 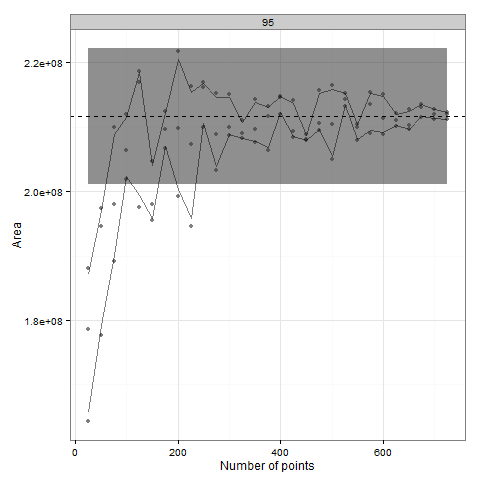 | 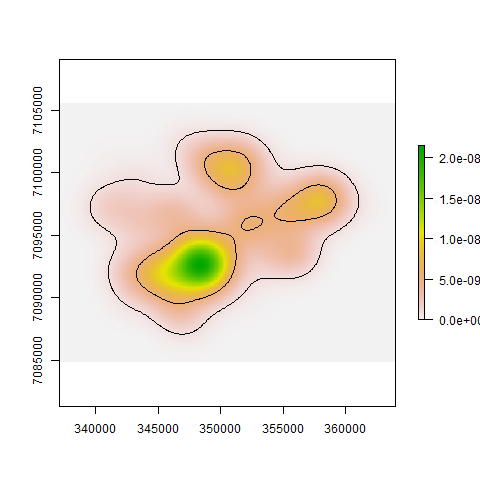 |
| 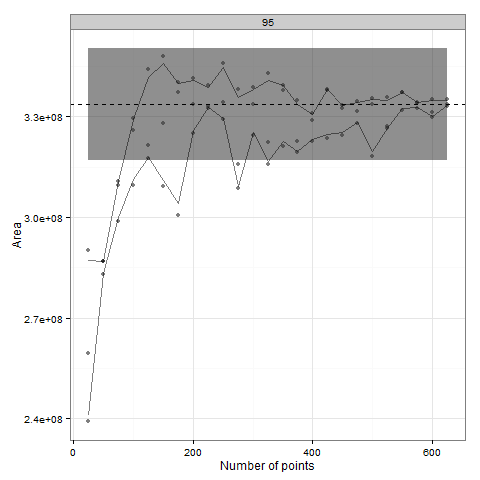 | 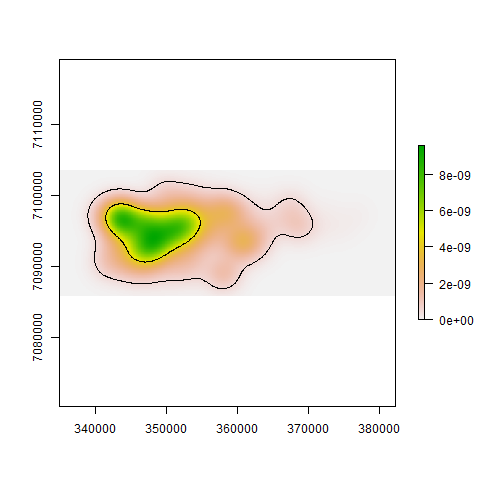 |
| 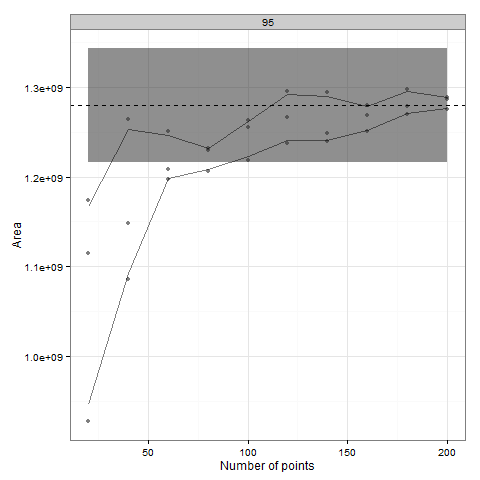 | 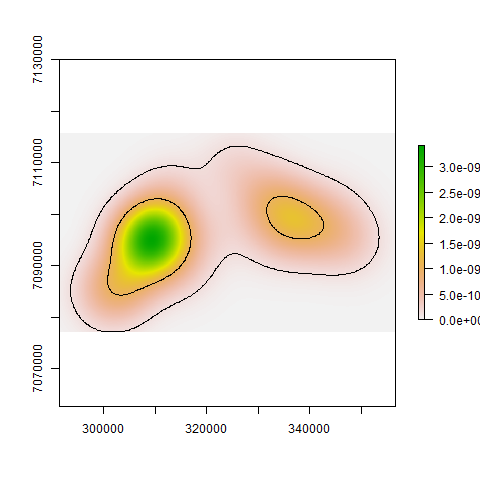 |
| 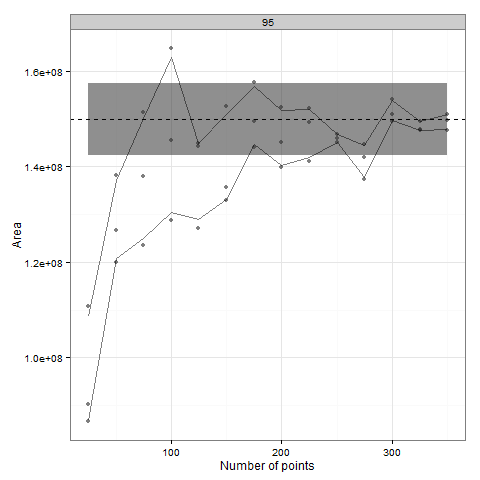 | 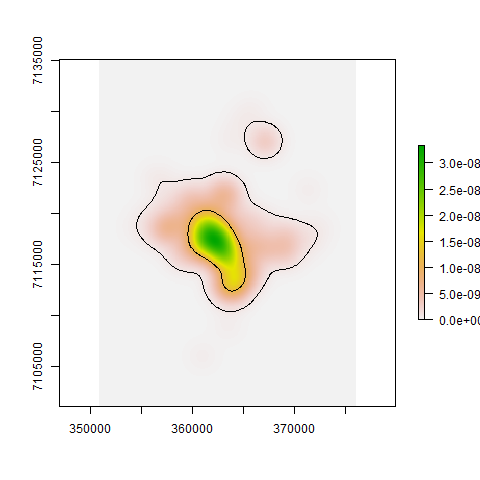 |
| 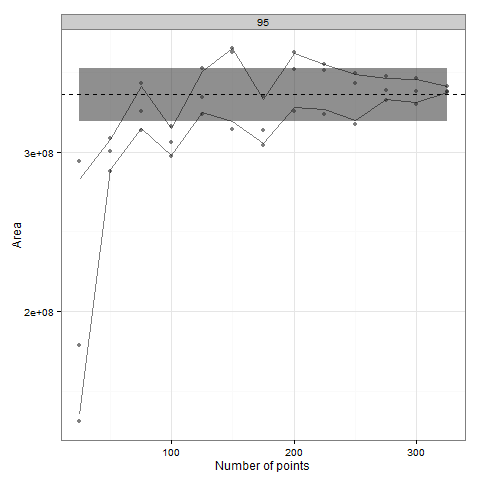 | 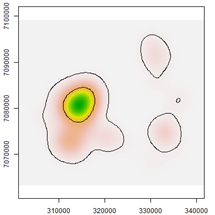 |
